# The burden of traumatic brain injury on caregivers: exploring the predictive factors in a multi-centric study

**DOI:** 10.1186/s40359-024-01652-6

**Published:** 2024-03-15

**Authors:** Mehran Ilaghi, Farhad Gharib, Ahmad Pirani, Abdol-Hossein Vahabie, Jordan Grafman, Seyed Vahid Shariat, Behnam Shariati, Amin Jahanbakhshi, Fatemeh Sadat Mirfazeli

**Affiliations:** 1 Institute of Neuropharmacology, Kerman Neuroscience Research Center, Kerman University of Medical Sciences, Kerman, Iran; 2 Mental Health Research Center, Psychosocial Health Research Institute, School of Medicine, Iran University of Medical Sciences, Tehran, Iran; 3 School of Electrical and Computer Engineering (ECE), College of Engineering, University of Tehran, Tehran, Iran; 4 Faculty of Psychology and Education, University of Tehran, Tehran, Iran; 5 School of Cognitive Sciences, Institute for Research in Fundamental Sciences (IPM), Tehran, Iran; 6 Shirly Ryan AbilityLab, Departments of Physical Medicine and Rehabilitation, Neurology, Cognitive Neurology, and Alzheimer’s Center, Chicago, IL USA; 7 Department of Psychiatry, Feinberg School of Medicine, Department of Psychology, Weinberg College of Arts and Sciences, Northwestern University, Chicago, IL USA; 8 Mental Health Research Center, Department of Psychiatry, School of Medicine, Psychosocial Health Research Institute, Iran University of Medical Sciences, Tehran, Iran; 9 Skull Base Research Center, Iran University of Medical Sciences, Tehran, Iran; 10 National Brain Centre, Iran University of Medical Sciences, Tehran, Iran

**Keywords:** Traumatic brain injury, Caregiver burden, Family burden

## Abstract

**Background:**

Traumatic brain injury (TBI) is a significant cause of mortality and morbidity worldwide. With survivors often exhibiting degrees of function loss, a significant burden is exerted on their caregivers. The purpose of this study was to explore the predictive factors of caregiver burden among caregivers of patients with TBI.

**Methods:**

Sixty-eight family members of individuals with a TBI who had been admitted to three hospitals were assessed in terms of caregiver burden using the Zarit Burden Interview. The association of caregiver burden with patients’ baseline cognitive function according to the Montreal Cognitive Assessment (MoCA) test, as well as caregivers’ sociodemographic characteristics, were evaluated using multiple regression analysis.

**Results:**

Based on the multiple regression model, the MoCA score of the patients (std β=-0.442, *p* < 0.001), duration of caregiving (std β = 0.228, *p* = 0.044), and higher education of the caregivers (std β = 0.229, *p* = 0.038) were significant predictors of caregiver burden.

**Conclusion:**

Overall, our findings highlight the importance of taking caregivers’ psychosocial needs into account. Long-term caregivers of TBI patients with cognitive impairment should be viewed as vulnerable individuals who could benefit from psychosocial intervention programs, to improve their well-being and enabling them to enrich their care of the TBI patient.

## Background

Traumatic brain injury (TBI) is an acquired injury to the brain resulting from any external physical force transmitted to the head [1]. It is often referred to as a silent epidemic, with almost 70 million new cases annually worldwide [2]. The epidemiology of TBI is diverse, with more cases occurring as a result of motor vehicle traffic accidents in developing countries and an increase in falls among the elderly in developed countries as the population ages [3]. In Iran, trauma is reported as the second leading cause of death, and TBI is considered a serious complication of traumatic events, particularly due to the high number of traffic accidents [4, 5].

Previously, high mortality rates due to TBI were unavoidable; however, advances in acute trauma care have secured a drop in mortality rates [6]. On the other hand, with an increased number of survivors and TBI-related functional impairments [7], patients with TBI frequently experience temporary or long-term motor and cognitive deficits, necessitating the provision of responsive nursing care around-the-clock [8].

The major responsibility of caring for these patients is often placed on the shoulders of a family member who acts as the primary caregiver. The needs of the caregiver, who plays a crucial role in the care of a patient with a TBI, are frequently overlooked and disregarded, resulting in an increased psychosocial burden on the caregiver [9]. Caregiver burden is conceptually defined as “the extent to which caregivers perceive that caregiving has had an adverse effect on their emotional, social, financial, physical, and spiritual functioning” [10].

The dynamics that alter caregivers’ psychological, behavioral, and emotional burdens, as well as interventions that are effective for the caregivers’ well-being, have received attention in recent studies [11–13]. Building on the transactional model of stress and coping, caregivers often face a multitude of stressors, ranging from the emotional toll of witnessing the decline in the health of their loved ones to the practical challenges associated with caregiving responsibilities, which necessitates the appraisal of stressors and applying appropriate coping strategies [14]. Previous studies have highlighted the detrimental effects of caregiver burden on caretakers, including increased depression, anxiety, disrupted sleep, and reduced quality of life [15–17]. Fewer studies have aimed to address the predictive factors of caregiver burden, mostly in neurodegenerative diseases such as Alzheimer’s disease. For instance, a variety of factors, including neuropsychiatric symptoms, the severity of the patient’s dementia, socioeconomic status, and the age of the caregiver, have been suggested as predictors of caregiver burden [18, 19].

In the case of TBI, recent studies suggest that both functional disability and caregiver-associated factors, including time spent caregiving, could be associated with caregiver burden [20]. However, there is a paucity of research on predictors of burden among caregivers of TBI patients in Iran. When taken as a whole, identifying vulnerable caregivers offers the opportunity to support them in order to avoid any potential long-term effects that may result from caregiver burden. Therefore, in this multi-center study in Iran, we investigated the predictive factors of caregiver burden among patient-related factors, such as cognitive function, as well as socio-demographic factors related to the caretakers.

## Methods

### Study design and participants

The present study was a cross-sectional study that examined if there were predictors of caregiver burden in caregivers of patients suffering from TBI. The study population consisted of family members of TBI patients who had been admitted to three academic hospitals of the Iran University of Medical Sciences (Firoozgar Hospital, Rasoul Akram Hospital, and Haft-Tir Hospital) in Tehran, Iran between June-August 2021. For each patient, the family member who was mostly involved in the direct care of the patient was included in the study. Individuals were excluded, if the responsibility of their patient care was entrusted to a nurse, or if they had transferred their patients to a rehabilitation center. Through a census method, all TBI patients during the study period were contacted. Of the initial 115 evaluated patients who were diagnosed with TBI, a total of 68 family members (20 males and 48 females, aged 18 to 65 years old) consented to participate in the study.

### Data collection procedure

Patients and their family members were contacted by a trained interviewer during their follow-up visits to rehabilitation centers. The objectives of the study were clearly outlined, and informed consent was obtained. The demographic data of caregivers, including age, gender, marital status, education level, job, place of residence, economic status, their relationship to the patient, and the demographic data of the patients were recorded. The cognitive function of the patients was assessed using the Montreal Cognitive Assessment (MoCA) during their first post-discharge visit to the clinic. To assess caregiver burden, the caregivers were asked to complete the self-report Zarit Burden Interview. For participants lacking reading literacy, the interviewer read the questionnaire items aloud to facilitate their participation in the study.

### Measures

#### Montreal cognitive assessment (MoCA)

MoCA is a cognitive screening test initially developed for mild cognitive impairment in degenerative central nervous system diseases and has previously been used to assess cognitive impairment in TBI as well [21]. The Persian version has been validated in previous studies [22]. This test contains several aspects of cognitive functions, including visuospatial/executive function (5 points; items such as alternate trail making, drawing a clock, and copying a cube structure), naming (3 points; naming the shapes of three animals), memory and delayed recall (5 points; repeating a list of five words, and recalling them in two trials), attention (6 points; items including reading a sequence of numbers forward and backward), language (3 points; items such as repeating a sentence and assessment of verbal fluency), abstraction (2 points; items including a statement of the similarities between two words), and orientation to time and place (6 points; stating the exact date, place, and city). The total score on the scale is 30, where higher scores indicate better cognitive function. It has been suggested that a MoCA score of 18–25 represents mild cognitive impairment, while scores 10–17 and less than 10 account for moderate and severe cognitive impairments, respectively [23].

#### Zarit burden interview

Zarit Burden Interview is the most established self-report measure to assess caregiver burden, which was originally developed by Zarit et al. [10]. This scale is being widely used as a measure of subjective caregiver burden due to its validity, reliability, brevity, ease of administration, as well as its translation and cultural adaptations in several languages. The scale focuses on aspects of the caregiver’s perceived burden, including the caregiver’s health, finances, social life, psychological well-being, and the relationship with the patient. In the current study, we used a validated 29-item Persian version of the scale with cultural adaptations [24]. Some instances of the items include “Do you feel that your patient’s demand for help is more than his/her real needs?”, “Do you feel that you do not have enough time for yourself because of the time you spend on your patient?”, “Do you feel stuck between caring for your patient and fulfilling your other family or work responsibilities?”. The scale is scored based on a 5-point Likert scale (never = 0 to nearly always = 4), where the total score is 116, and a higher score demonstrates a higher burden [24].

### Statistical analysis

All analyses were performed using the Statistical Package for the Social Sciences (SPSS) software (version 22.0. SPSS, Inc., Chicago, IL, USA). The categorical variables were described using the terms frequency and percentage, and the continuous variables utilized the mean (± SD). A bivariate Pearson correlation analysis was used to assess the correlation of quantitative variables with caregiver burden score. To identify whether there were factors predicting caregiver burden, a multiple linear regression analysis was used. The unstandardized and standardized regression coefficients were reported in the final model. A *p* < 0.05 was used to denote statistical significance.

## Results

A total of 68 caregivers participated in the study, of whom 70.6% were female. The mean (± SD) age of participants was 42.22 (± 11.19) years ranging from 18 to 65 years. The majority were married (91.2%) and had only completed primary school (38.2%). 83.8% of the participants lived in urban areas. Most participants had either moderate (48.5%) or low (47.1%) socioeconomic status and were housewives (48.5%) or freelance workers (38.2%). Most caregivers (55.9%) were the spouse of the patient. The mean (± SD) duration of caregiving since the TBI was 10.22 (± 7.45) months. Detailed characteristics of the caregivers are presented in Table 1 (Table 1).

**Table 1:** Demographic characteristics of the participants

Variables		N(%)
**Gender**	Male	20 (29.4)
	Female	48 (70.6)
**Marital status**	Single	6 (8.8)
	Married	62 (91.2)
**Education**	None	6 (8.8)
	Primary school	26 (38.2)
	Secondary/High school	3 (4.4)
	Diploma	25 (36.8)
	University degree	8 (11.8)
**Place of residence**	Urban	57 (83.8)
	Rural	11 (16.2)
**Socioeconomic status**	Low	32 (47.1)
	Moderate	33 (48.5)
	High	3 (4.4)
**Job**	Unemployed	1 (1.5)
	Housewife	33 (48.5)
	Employed	6 (8.8)
	Freelance worker	26 (38.2)
	Student	2 (2.9)
**Relationship with the patient**	Father	11 (16.2)
	Mother	12 (17.6)
	Sibling	3 (4.4)
	Spouse	38 (55.9)
	Son/Daughter	4 (5.9)
		**Mean (± SD)**
**Age (years)**		42.22 (± 11.19)
**Duration of caregiving (months)**		10.22 (± 7.45)

Considering that the total score on the MoCA scale is 30, and the higher scores indicate better cognitive function, the mean (± SD) score of the MoCA in the TBI patients was 17.56 (± 5.54), ranging from 3 to 27 (Table 2). Based on the severity cut-off threshold of MoCA scale, 7.4% of the patients did not have cognitive impairment. However, 45.6% of the patients suffered from mild cognitive impairment (score 18–25), while moderate (score 10–17) and severe (score < 10) cognitive impairment was observed in 36.8% and 10.3%, respectively. Additionally, considering that the total score on the Zarit Burden Interview is 116 and higher scores represent higher burden, the mean (± SD) score of caregiver burden among our studied caregivers was 47.94 (± 21.11), ranging from 9 to 94 (Table 2).

**Table 2:** Cognitive function assessment of TBI patients according to the MoCA test as well as the caregiver burden according to the Zarit Burden Interview

Instrument	Minimum score obtained	Maximum score obtained	Mean Score	Standard deviation
**MoCA**	3	27	17.56	5.54
**Zarit Burden Interview**	9	94	47.94	21.11

A bivariate Pearson correlation analysis demonstrated that the duration of caregiving (*r* = 0.388, *p* = 0.002), age of the caregiver (*r*=-0.242, *p* = 0.047), and the MoCA score of the patient (*r*=-0.495, *p* < 0.001) were correlated with the caregiver burden score. However, when the potential predictiors were assessed in the multiple linear regression analysis using a backward method, the final model revealed that the MoCA score (standardized β=-0.442, *p* < 0.001), duration of caregiving (standardized β = 0.228, *p* = 0.044), and higher education of the caregiver (standardized β = 0.229, *p* = 0.038) were determinants of caregiver burden (Table 3). This model explained 31.9% of the variance of the dependent variable (R^2^ = 0.319).

**Table 3:** Predictors of caregiver burden in multiple linear regression model

Independent variable	Unstandardized B coefficient	Std. Error	Standardized β coefficient	t	Sig.
**(Constant)**	62.970	9.448	-	6.665	0.000
**Duration of caregiving**	0.693	0.337	0.228	2.057	0.044
**MoCA score**	-1.612	0.403	− 0.442	-3.996	0.000
**Education (non-illiterate)**	6.502	3.067	0.229	2.120	0.038

## Discussion

To the best of our knowledge, this is the first study assessing the predictive factors of caregiver burden among caretakers of TBI patients in Iran. The majority of TBI caregivers in our Iranian study were married females with low education levels, and the mean duration of caregiving in our study population was nearly ten months. Overall, the findings of the current study indicated that the cognitive function of the patient, the education level of the caregiver, and the duration of caregiving were associated with the caregiver burden.

Our findings revealed that the baseline MoCA score of the patient was inversely associated with caregiver burden, indicating that the better the cognitive function of the TBI patient is, the lower the burden on the caregiver will be. This finding is consistent with the burden reported by caregivers of patients with neurodegenerative disorders. For instance, Klietz et al. reported that the MoCA score could be a predictive factor of caregiver burden in caregivers of advanced Parkinson’s disease patients [25]. Similar findings have been demonstrated in several studies on caregivers of patients with Alzheimer’s disease [26, 27] or Huntington’s disease [28]. In patients with TBI, it has previously been demonstrated that TBI-related brain lesions that affect the cognition of patients are associated with greater long-term burden on the caregivers [29]. Moreover, cognitive decline in TBI survivors has been known to significantly affect the caregiver burden [30]. These findings could be explained by the fact that cognitive disabilities are occasionally linked with a reduction in the patient’s independence and interference with daily functioning. Therefore, a higher level of disability may necessitate more support from the caregiver, which contributes to a heightened level of caregiver burden. Moreover, according to the transactional model of stress and coping [14], caregivers facing higher levels of cognitive impairment in their patients, may encounter increased stress due to the dynamic interplay of appraisal processes and coping mechanisms. The escalating demands associated with managing complex care needs, communication challenges, and behavioral changes contribute to the heightened caregiver burden. These results underscore the need for both physical and cognitive function rehabilitation, which are not only crucial for the patient but could potentially ameliorate the burden on the caregiver [20]. In terms of TBI, numerous rehabilitation interventions have been proposed. In a comprehensive scoping review, Sveen and colleagues have gathered interventional TBI rehabilitation studies, reporting that these interventions encompass a broad spectrum of hospital and community/home rehabilitations with a variety of focuses, including daily life, work/education, emotional factors, and cognitive deficits [31]. Therefore, providing care recipients with more rehabilitation interventions, aiming to enhance cognitive function or to decelerate cognitive decline could increase their independency and thereby lessen the burden placed on their caregivers.

The results of our study also revealed that the higher education level of the caregiver was a predictor of caregiver burden. However, the findings of the previous studies have not been conclusive in this regard. For instance, Vahidi et al. reported that a lower educational level placed more burden on caregivers, suggesting that those with higher education use problem-focused coping skills in dealing with difficulties of caregiving [32]. Similar findings have been observed in a study by Kim et al. [33]. On the other hand, in line with our findings, Sander and colleagues reported that higher education level was associated with an increased burden on caregivers of patients with TBI [34]. They attributed this observation to the fact that well-educated individuals tend to set unrealistic expectations regarding their caregiving goals and often show reluctance to reach out for support. Similarly, Schnitzer et al. reported that better-educated caregivers had higher odds of perceiving increased burden [35]. According to the authors, those with higher education might be more concerned about losing their autonomy, thereby perceiving more burden. Overall, further research is required to undermine the role of a caregiver’s education level in determining caregiver burden.

Not surprisingly, our findings also demonstrated that the duration of caregiving was a significant predictor of caregiver burden in patients with TBI, suggesting cumulative burden of caregiving. In line with this finding, in a study by Doser et al. on caregivers of patients with severe brain injury, it was indicated that caregivers who spent more time taking care of their loved ones had a higher level of burden [36]. Similarly, Jaracz and colleagues reported that the burden on caregivers of long-term stroke survivors was independently associated with the amount of time spent caregiving [37]. Additionally, Lou et al. demonstrated that a longer duration of being a caregiver was associated with higher levels of burden among caregivers of patients with Alzheimer’s disease [38]. Moreover, as our findings imply, the majority of caregivers were married and freelance workers with low to moderate socioeconomic status. These individuals are often highly overwhelmed with financial activities and the act of caregiving might interfere with their daily activities and negatively impact their economic status. As the role strain theory posits, as the caregiving role persists, individuals may encounter increasing challenges in fulfilling their caregiving duties, resulting in elevated burden levels [39, 40]. These observations underscore the fact that caregivers who have been engaged with caregiving for a longer time are more vulnerable and in need of both psychological and financial support.

Our study had its strengths and limitations. This study was a multicentric study consisting of participants with different demographic and socio-economic backgrounds in three distinct regions of Tehran. Therefore, the potential selection bias that could occur due to the type of hospital was minimized. However, some reporting bias might exist as the introduction of the person with the most contribution to patient care was subjectively reported by the family members. Therefore, in families whose patient care was divided among caregivers, the burden was not measured in all the caregivers. Accordingly, further studies may be needed to assess how the increased number of caregivers and the perceived social support of each caretaker might affect the burden on individuals within the caretaker group.

## Conclusions

Overall, this study showed that the MoCA score of the patients, the higher education of the caregivers, and the duration of caregiving were significant predictors of caregiver burden. Our study highlights the relevance of taking caregivers’ psychosocial needs into account. Long-term caregivers of TBI patients with higher cognitive impairments should be viewed as particularly vulnerable individuals who could benefit from intervention programs, both to improve their well-being and to improve the patient’s functionality, thus alleviating the burden on their caregivers. Lastly, our findings in Tehran demonstrate the cross-cultural similarities including degree of burden facing caregivers of TBI patients.

## Data Availability

The data analyzed during the current study are available from the corresponding author upon reasonable request.

## Abbreviations

TBI: Traumatic Brain Injury
MoCA: Montreal Cognitive Assessment
